# Improved electro-mechanical performance of gold films on polyimide without adhesion layers

**DOI:** 10.1016/j.scriptamat.2015.02.005

**Published:** 2015-06

**Authors:** Barbara Putz, Rachel L. Schoeppner, Oleksandr Glushko, David F. Bahr, Megan J. Cordill

**Affiliations:** a Department of Materials Physics, Montanuniversität Leoben, Jahnstrasse 12, Leoben 8700, Austria; b Erich Schmid Institute of Materials Science, Austrian Academy of Sciences, Jahnstrasse 12, Leoben 8700, Austria; c School of Mechanical and Materials Engineering, Washington State University, Pullman, WA 99164, United States; d School of Materials Engineering, Purdue University, West Lafayette, IN 47907, United States

**Keywords:** Thin films, Mechanical properties, Electrical resistivity, Fracture, Fragmentation

## Abstract

Thin metal films on polymer substrates are of interest for flexible electronic applications and often utilize a thin interlayer to improve adhesion of metal films on flexible substrates. This work investigates the effect of a 10 nm Cr interlayer on the electro-mechanical properties of 50 nm Au films on polyimide substrates. Ex situ and in situ fragmentation experiments reveal the Cr interlayer causes brittle electro-mechanical behaviour, and thin Au films without an interlayer can support strains up to 15% without significantly degrading electrical conductivity.

## Graphical abstract

**Figure:**
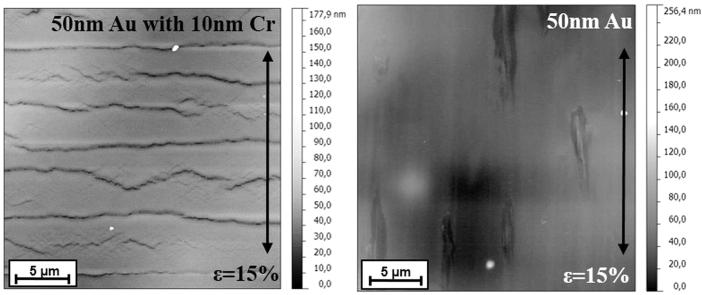

  Flexible electronic devices, consisting mainly of thin metal films on compliant polymer substrates, are of great scientific and industrial interest [1,2]. The usability of a flexible electronic device rises and falls with the electro-mechanical performance of the metal film/polymer substrate couple. Straining metallic conductors to large deformations while maintaining a low and constant electrical resistance is one of the main challenges for flexible electronics technologies [3,4]. “Stretchability,” the ability of a film/substrate system to support large stretching, during use, without failing mechanically or electrically, is a keyword used to describe the desired electro-mechanical behaviour of flexible thin film systems. In order to get a high stretchability, a high fracture strain and good adhesion between the metal film and the substrate are required [4]. To achieve high fracture strains methods such as film structuring [5] and wrinkling [6,7] have been utilized. A common way to improve the adhesion of the ductile conductive metal layers such as Au is to use 5–10 nm of brittle Cr, Ti or Ta as adhesion layers [8,9]. However, it is known that the deformation behaviour of ductile and brittle thin metal films on compliant polymer substrates strongly differs from each other [8,10]. Brittle metals used as adhesion promoters will fracture at low applied strains (<1%) leading to electrical failure, while ductile charge carrying films (Au, Cu, Ag) plastically deform at higher applied strains (>3%) without electrical failure. The ability to combine good adhesion and high strength for these flexible systems is the current challenge.

This work utilizes fragmentation testing [11–14] and in situ 4 point probe resistance measurements (in situ 4PP) [3,5,15,16], both under uniaxial tensile strain, to determine the effect of a 10 nm Cr adhesion layer on the electro-mechanical behaviour of 50 nm Au films on a flexible polyimide substrate and to assess the suitability of these film systems for flexible electronic technologies. It will be shown that the use of brittle adhesion layers, while possibly promoting adhesion, actually decrease the electrical performance by inducing the formation of cracks in the ductile layer.

Ductile 50 nm Au films were sputter deposited onto 50 μm thick flexible Kapton polyimide (PI) substrates after cleaning with isopropyl alcohol. To improve the adhesion of the Au film, a 10 nm Cr layer was deposited before the Au film without breaking vacuum. For Au films without a Cr layer Au was deposited directly onto PI. Sputtering was performed with a DC Magnetron system and the deposition parameters for the 10 nm Cr layer were a working gas (Ar) pressure of 1.5 × 10^−3^ Torr at 100 W DC power. Sputtering of the Au film was performed at a working gas (Ar) pressure of 7.5 × 10^−3^ Torr at a power of 75 W DC. The Au films had a nanocrystalline microstructure with a grain size between 20 and 50 nm measured using transmission electron micrographs [17]. Rectangular samples used for tensile straining were cut with a scalpel to the dimensions of 7 × 35 mm.

Fragmentation testing was carried out using an Anton Paar TS600 straining stage. Samples were strained to different maximum strains equal to % *ɛ* = 2, 5, 10, 12 and 15 with a constant displacement rate of 5 μm/s at room temperature and humidity. The in situ 4PP measurement was performed with an MTS Tyron250® universal testing machine following the process of Glushko et al. [15]. Four samples of each film system were strained to a maximum strain of 15% and unstrained with a constant displacement rate of 5 μm/s. The contact resistance of the film under the straining grips was determined with the aid of a second 4PP setup prior to electro-mechanical testing and subtracted from the measured values in order not to underestimate the resistance increase during straining [15]. After straining the sample surfaces were imaged with an atomic force microscope (AFM) and a scanning electron microscope (SEM) to quantify the amount of mechanical damage as a function of strain. Selected strained Au/Cr samples were etched with *aqua regia* (3 parts 32% HCl, 1 part 65% HNO_3_) to remove the Au film in order to compare the cracking in the Cr layer to the Au film.

AFM and SEM images were analysed with GWYDDION [18] and ImageJ [19], respectively. The average crack spacing, *λ*, (distance between through thickness cracks; TTCs) of each strained sample was measured following the process reported by Cordill and Marx [20]. Figure 1a and b shows an AFM height image at 15% strain and the extracted height profile. With the surface profiles TTCs were identified (black arrows). A Δ*/h* ratio > 10% (Δ = deformation or crack depth, *h* = film thickness) [20] was applied to distinguish between TTCs and film necking *(*Δ/*h* < 10%). For SEM images it was assumed that every crack visible in the micrograph was a TTC (black arrows, Fig. 1c). Average values of the crack spacing as well as the standard deviation were calculated for each sample.

The Au films with the Cr interlayer formed TTCs perpendicular to the straining direction under uniaxial tension (Fig. 1a and c). Cracks were not observed in the Au films without the Cr interlayer within the tested strain regime. Therefore, a crack spacing analysis was only performed for Au/Cr films. Figure 1d shows the crack spacing evolution in the Au/Cr films as a function of applied strain for the AFM and SEM analysis. Because of the brittle behaviour of the film system the two analyses closely follow one another. Furthermore, the assumptions that every visible crack in the SEM images is a TTC and the Δ/*h* ratio >10% from the AFM analysis are valid. TTCs form at low strains (2% strain) and the crack spacing saturation, indicated by a plateau in Figure 1d, also occurs at low strains (saturation strain *ɛ_s_* ∼ 4%). When the crack spacing reaches a plateau with increased strain, no further cracking of the film occurs and cracks only open due to stretching of the polymer substrate. At this plateau, the saturation crack spacing can be determined as *λ*_s_ = 4.3 ± 1.4 μm. Interestingly, buckle delamination between the crack fragments was not observed even after straining 15%. This is an indication that the Cr adhesion layer could be improving the adhesion between the metal films and polymer substrate, however, the presence of TTCs would infer that the electrical behaviour of the films is severely decreased after straining.

Figure 2 shows the recorded electrical resistance for the Au films with and without the Cr interlayer, normalized by the initial resistance, *R*_0_, as a function of the relative elongation and the theoretical curve, *R*/*R*_0_ = (*L*/*L*_o_)^2^. This relation is applicable as long as there are no changes in resistivity and volume conservation is satisfied during the experiment [15]. However, when a film exhibits substantial structural modifications such as cracks, the resistance deviates from this theoretical line. What can be clearly observed in Figure 2 is that the Au film follows the theoretical behaviour up to the maximum prescribed strain, while the Au/Cr film deviates from the theoretical curve due to TTC formation. Despite crack formation in the Au/Cr film, complete electrical failure was not observed at a maximum strain of 15% (not shown in Fig. 2 for clarity). The maximum normalized resistance at 15% strain *R*/*R*_0_*_max_* was more than 8 times higher than the initial normalized resistance. Individual cracks propagate through the thickness of the metal layer but not over the whole width, leaving bridges for electrons to flow (Fig. 3a). During unloading the resistance decreases with the same slope as the crack-edges become contacted again. The final resistance *R_end_* is about 50% higher than the initial resistance indicating the presence of cracks. However, the preservation of electrical conductivity provides evidence of the presence of crack bridges (Fig. 3a).

In contrast, the Au films without the Cr interlayer showed outstanding electro-mechanical performance. The relative resistance followed the theoretical curve very closely, with *R*/*R*_0_ increasing only because of a change in the sample dimensions during straining with no deviation indicating fracture of the film. The final resistance *R_end_* was approximately the same as the initial resistance. AFM observations of the sample surfaces before and after straining 15% confirmed that there was no localized necking or cracks perpendicular to the straining direction (Fig. 3b). The features parallel to the straining direction stem from the deposition process. Compared to Au/Cr wrinkled films on elastomers [6,7] and structured films [5], the stretchability of the 50 nm Au films on PI should be considered to be significant. The Au-PI system can withstand a large amount of strain without observable deformation or fracture without the aid of pre-straining before deposition or film structuring. Furthermore, the Au film without the Cr adhesion layer did not delaminate; indicating that the adhesion of the Au-PI interface is just as strong, if not stronger than the Cr-PI interface. Similar electrical results have been observed with wrinkled Au/Cr films and lines on elastomer substrates [6,7]. With wrinkling, the system can be strained to macroscopic strains of 20% without significant loss of the electro-mechanical integrity. The Au-PI system described here achieves similar strains, but without relying on out of plane deformation to reach macroscopic in-plane extension. These parallels in the electro-mechanical behaviour between wrinkled films and non-wrinkled films show the long range effect of brittle adhesion layers on stretchability.

The ex situ fragmentation results correlate well to the in situ resistance measurements. Figure 4 shows a comparison of the ex situ crack spacing and the in situ electro-mechanical data for Au/Cr films. The in situ 4PP and ex situ crack results correspond well and are within the experimental error. The deviation of the resistance from the theoretical curve at about *ɛ_f_* = 2.5% in the in situ 4PP experiment occurs after the cracking onset during ex situ fragmentation testing at 2% strain, suggesting the presence of cracks does not significantly alter the electrical behaviour until a certain crack density has formed and saturated (between 2 and 5% strain in this case). For ductile Au films no deviation of the resistance from the theoretical curve corresponds to the absence of crack formation after straining.

The cracks in the Cr adhesion layer correspond directly to the cracks observed on the surface of the Au film, as shown in the inset of Figure 4. The crack spacing in the Au film is 4.9 ± 0.4 μm and in Cr film is 4.8 ± 0.6 μm, which leads to the conclusion that the Cr film causes brittle fracture of a ductile Au film. A similar cracking behaviour has recently been observed in Cu films (50–200 nm) with a 10 nm Cr interlayer [21] where TTC was observed for all Cu/Cr films but not for Cu films of the same thicknesses without the Cr layer. These results were modelled with finite elements and found that the cracks form initially in the Cr layer and act as stress concentrators in the Cu film [21]. For films greater than 200 nm the effect could be difficult to observe without examining a large strain range (>20%), however, the brittle interlayer will still fail first as shown in [8,21].

Ex situ fragmentation testing and in situ 4PP experiments revealed that a 10 nm Cr adhesion layer causes brittle electro-mechanical behaviour of normally ductile 50 nm Au films as shown by the correlation between ex situ and in situ fragmentation results. In the Au/Cr film, cracks form perpendicular to the straining direction between 2 and 2.5% strain and after initial fracture, crack spacing saturation occurs at approximately 4% strain. The average measured saturation crack spacing was *λ*_s_ = 4.3 ± 1.4 μm. Although the resistance increased eightfold during straining, complete electrical failure of the Au/Cr film system was not observed at a maximum strain of 15%. However, the sharp resistance increase after the initial fracture at only 2.5% strain is unacceptable for electric conductors in flexible devices and the system was considered “failed” in terms of suitability for flexible electronic applications. In contrast, the 50 nm Au films without the Cr adhesion layer showed very ductile behaviour under uniaxial tension and outstanding electro-mechanical performance. No evidence of film cracking or localized deformation that may be linked to delamination or necking was observed up to a maximum strain of 15%. The results of the experiments presented here indicate that adhesion layers may not be necessary to improve the adhesion of ductile films to all polymers. Rather, the deposition method and resulting film and interface structure may be the dominant parameters controlling stretchability of metal-polymer systems.

## Figures and Tables

**Figure 1:**
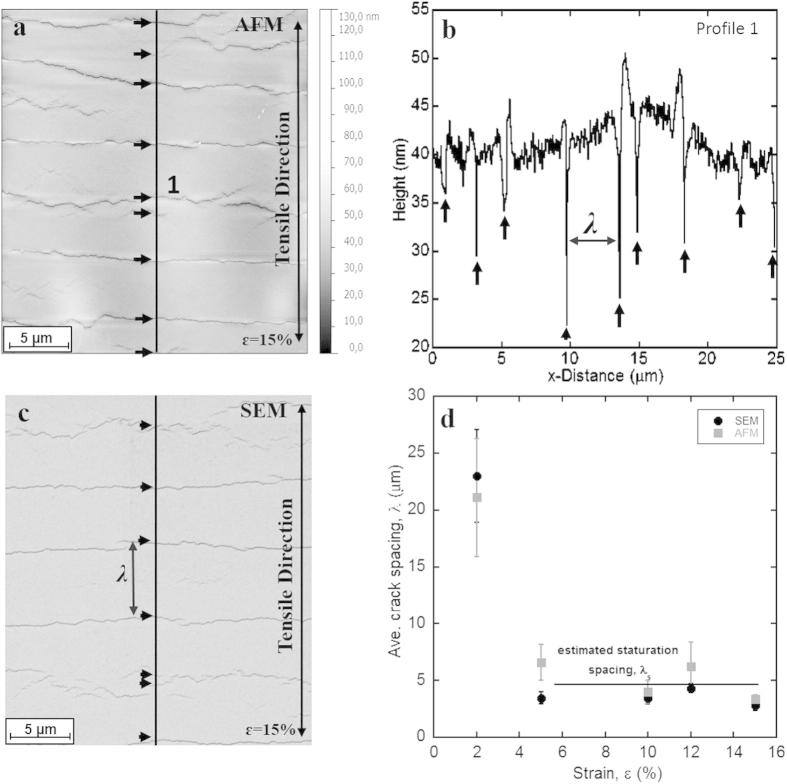
(a) AFM height image of Au/Cr after 15% strain and (b) corresponding surface profile. (c) SEM micrograph of Au/Cr after 15% strain. Straining directions are indicated. Black arrows indicate TTCs and *λ*. (d) Average crack spacing evolution as a function of strain measured with AFM and SEM. The standard deviation is depicted with error bars. The estimated saturation spacing is indicated by a straight line to guide the eye.

**Figure 2:**
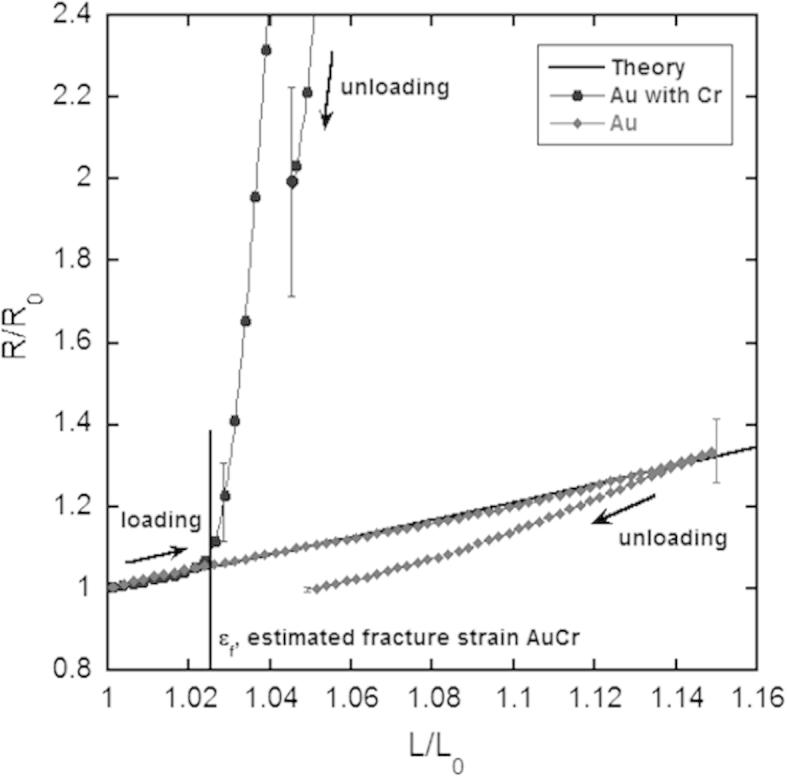
Experimental normalized resistance *R*/*R*_0_ as a function of uniaxial elongation for Au films with and without a Cr interlayer plotted with the theoretical resistance assuming volume conservation and constant resistivity during deformation. Error bars depict standard deviation of four experiments at representative points.

**Figure 3:**
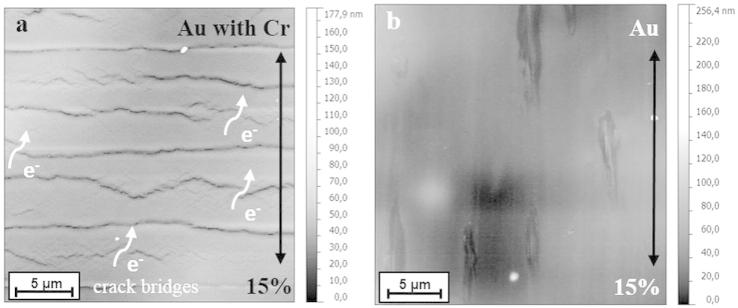
AFM height images of (a) Au with Cr and (b) Au films strained 15%. Straining direction is indicated by the arrows. The features parallel to the straining direction in (b) stem from deposition and are not due to straining.

**Figure 4:**
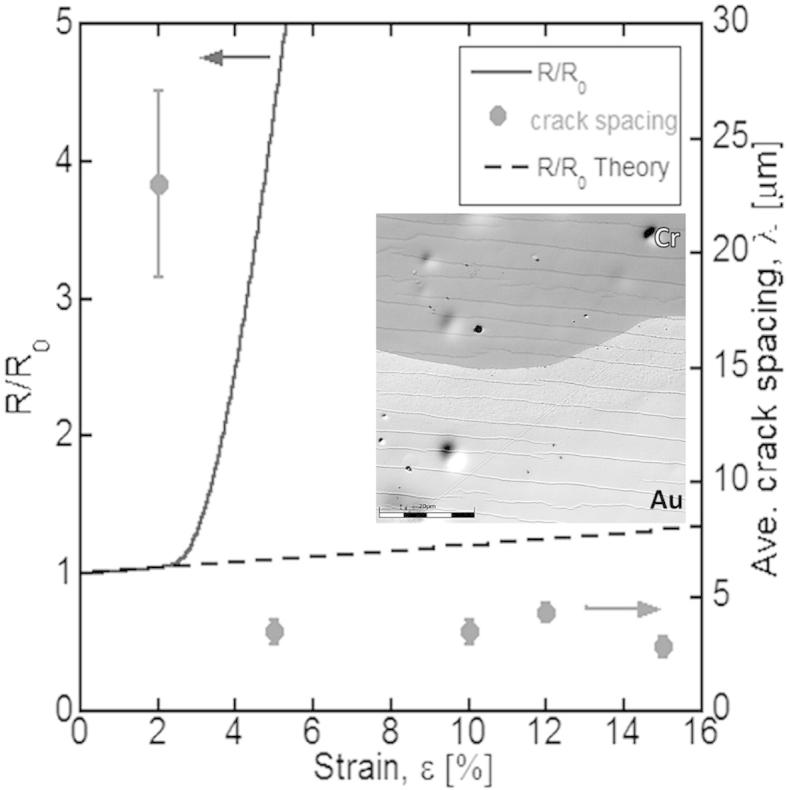
Comparison of in situ 4PP and ex situ fragmentation tests for Au/Cr films. The deviation from the theoretical electrical curve (dashed line) correlates with the fracture onset during the fragmentation test. Inset: scanning laser intensity image of the Au film partially etched away showing cracks in the Cr film corresponding to cracks in the Au film (scale bar 20 μm).
